# The Impact of Psychological Resources on Body Mass Index in Obesity Surgery Candidates

**DOI:** 10.3389/fpsyt.2020.00649

**Published:** 2020-07-10

**Authors:** Anita Robitzsch, Adam Schweda, Madeleine Hetkamp, Marco Niedergethmann, Nora Dörrie, Stephan Herpertz, Till Hasenberg, Sefik Tagay, Martin Teufel, Eva-Maria Skoda

**Affiliations:** ^1^ Department of Psychosomatic Medicine and Psychotherapy, University of Duisburg-Essen, LVR Clinic Essen, Essen, Germany; ^2^ Department of Surgery, Alfried-Krupp Hospital, Essen, Germany; ^3^ Department of Psychosomatic Medicine and Psychotherapy, LWL-University, Ruhr-University Bochum, Bochum, Germany; ^4^ Helios Obestiy Center West, Helios St. Elisabeth Hospital Oberhausen, Witten/Herdecke University, Helios Universitätsklinikum Wuppertal, Wuppertal, Germany; ^5^ Faculty of Applied Social Sciences, Technical University of Köln, Köln, Germany

**Keywords:** obesity surgery, psychological resources, body mass index, Eating Disorder Examination Questionnaire, Patients Health Questionnaire-d

## Abstract

**Background:**

Obesity surgery is the therapy of choice for severely obese patients. The results are promising, but at the same time obesity surgery represents a physical and psychological challenge for patients and care givers. In order to give psychosocial support adequately, more knowledge of effects of psychological profiles is required. Research is often deficit and symptom oriented. Psychological resources (competences) of individuals are often neglected. However, after surgery patients have to cope with the altered anatomic condition and therefore psychological resources are essential for a successful development and my influence also the surgical outcome. The interplay of eating behavior, depression, and psychological resources and their influence on weight are yet undetermined.

**Methods:**

A cross-sectional study in consecutive obesity surgery candidates was performed. One hundred twenty-seven participants were included (90 female, 37 male; mean BMI 49.85 kg/m²; range 36.7–84.2 kg/m²). After conducting semi-structured clinical interviews psychological resources, depression and eating behavior were assessed *via* three questionnaires: Essener Ressourcen-Inventar (ERI), Patient Health Questionnaire depression module (PHQ-d), and Eating Disorder Examination-Questionnaire (EDE-Q). To evaluate the influence of psychological resources on BMI mediation models and path analyses were performed.

**Results:**

Psychological resources do not influence BMI directly. Path analyses revealed depression as well as depression/eating behavior as mediating dimension. A first path showed that higher psychological resources are associated with less depressive symptoms and lower BMI. On the other side, a second path showed that higher psychological resources are related to less depression and by means of more conscious and controlled eating behavior to a lower BMI.

**Conclusion:**

Psychological resources seem to be relevant in the context of obesity surgery. Good psychological resources show plausible associations with less depression and a more adequate eating behavior. The evaluation of psychological resources in obesity surgery candidates allows the identification of patients at risk. Competences of patients should be addressed in the context of surgery. Our findings build a foundation for a more individualized supportive treatment for obesity surgery candidates. Improving impaired psychological resources may help in the coping process after surgery and is supposed to lead to an even higher weight loss.

## Introduction

Obesity is a globally increasing disease (1, 2). According to the WHO, obesity has nearly tripled since 1975. The current incidence is over 650 million adults worldwide (1). Patients with obesity often suffer from multiple somatic comorbidities such as diabetes, hypertension, and cardiac dysfunction (3–5). Treatments of these diseases are expensive and pose a burden not only to the patient but also to clinicians (6). The majority of the people worldwide live in countries where more people get killed by obesity and overweight than underweight. In 2016 over 340 million adolescents and children were overweight or obese (1). One major reason is a critical increase in daily caloric food supply; since the early 1980s, for instance, per capita caloric supply in Northern America increased by over 10% until 2017 (7). Similar developments can be observed all around the world (whereas such development is very convenient in poorer parts of the world). It is a mostly preventable disease that causes great avoidable costs every year (1). Over all obesity is a challenge not only for the individual but also for the society, such as raising health care costs, multiple comorbidities, and a shortened life expectancy (3).

In order to meet this challenge a variety of treatments exists. Beside conservative methods (e. g. diets, exercise, and lifestyle change) obesity surgery is a promising approach to not only help the severely obese person, but to also offer possibilities to lose weight to people with high levels of psychological suffering, or preexisting obesity-related co-morbidities whose impact can be attenuated *via* weight loss (8, 9).

Herpertz et al. (10) recently summarized several studies on psychosomatic and psychosocial questions regarding obesity surgery and pointed out the following: after a conservative weight loss program (“lifestyle interventions”) about 50% of the participants were able to reduce their weight by 5–10%. This is the minimal necessary weight loss to clinically significant reduce somatic risk parameters. Nevertheless, weight regain next to not losing weight at all appears to happen most of the time. Thirty percent to 50% of the people who initially lose weight regain it within 1 year of the end of treatment. Even after long-term treatments, only 4–5% maintain the initial weight loss. In the case of severely obese people (BMI ≥40 kg/m^2^) weight loss *via* a conservative approach is even lower and ranges between 2% and 6.9%.

Therefore, the Interdisciplinary European Guidelines on Metabolic and Bariatric Surgery recommend a surgical approach for patients in age groups from 18 to 60 years from a BMI ≥ 35 kg/m² (grade II obesity) onward, as soon as obesity-associated co-morbidities exist and conservative therapy options are exhausted (11). Up to this point, obesity surgery can be considered to be one of the most effective weight-loss interventions.

In addition to mostly promising results, however, different long-term outcomes (e.g. renewed weight gain after surgery or increased suicidal tendencies) can also be observed (12).

Obesity surgery candidates often have to face somatic and obesity-associated comorbidities. With regard to mental comorbidities obesity surgery candidates show increased levels of anxiety and depressive symptoms (13). Depression is also described as the most common psychiatric comorbidity (14). Furthermore, obesity surgery candidates tend to show increased pathological eating behavior and impulsivity (15). As with psychopathology in general, eating disorders are associated with elevated risk of suicidal behavior (9). Despite of the considerable amount of studies conducted, it stays rather unclear why some patients post obesity surgery tend to not lose weight or regain it (12, 16). Yet there seems to be a strong association with psychopathological aspects. So far, most approaches existing concentrate on analysis of symptoms and dysfunctions. The need for a more resource-oriented approach seems essential for a better understanding of the profile of patients’ psychological abilities, rather than simply to design a profile of their deficits. Therefore, it would be advisable to follow a more resource-orientated approach investigating psychological health issues and implement pre-operative support systems.

In this respect, psychological resources play a decisive role. The term psychological resources describes the totality of the protective and supportive competences used by a person or available to him and which enable external actions. Psychological resources are important predictors of mental health and important determinants of therapeutic success (17). In the sense of Klaus Grawe’s Consistency Theory (2004) (18), activation of psychological resources in psychotherapy patients is a central element of successful treatment. Psychological resource variables have a positive relation to the health level (19).

For example, Gerlach et al. (20) showed that patients with a comorbid personality disorder (PD) tend to lose less weight in conservative obesity weight-loss treatments than patients without PD. Therefore, the special needs of patients with comorbid PD should be more considered in weight-loss treatment strategies and prevention. Although there are findings that the existing of PD doesn’t attenuate weight loss post-surgery (21) it should be considered since Kalarchian et al. (22) found that 28.5% of obesity surgery patients suffer from PD.

Knowledge about psychological resources gives valuable information about the health level and allows targeted interventions. Psychological resources can be subdivided in personal, social and structural resources (23).

Central key are the personal resources (e.g. self-efficacy, self-acceptance, meaningfulness, openness/flexibility, positive handling of offenses, closeness to nature), which are regarded as enduring and central characteristics of personality. They provide the greatest variance explanation for predicting behavior in adults (24).

In addition, social resources (e.g. positive social bonds, practical support) (25, 26) and structural resources (e.g. financial security, environmental support) (27) also influence mental health. In order to a further comprehending what an individual needs and what skills he or she has to meet these needs, it is of utmost importance to better understand psychological resources.

This is also in line with studies regarding positive psychology and protective factors for health in general. Garaigordobil (28) for example showed that adolescents with more feelings of happiness had less pathological symptoms (e.g. somatization, depression) and that high self-esteem and high self-concept as well as few depressive symptoms are associated with prediction of happiness.

In summary, obesity is a rapidly spreading disease that affects individuals, society, and the global healthcare system (1), and obesity surgery is a well-established treatment option. Yet, not everyone profits equally, and some individuals are at risk of suffering severe difficulties and complications after the surgery (8). Studies show that mental illnesses such as depression and eating disorders can sabotage post-operative long-term weight loss (12, 13). To our knowledge, psychological resources were never taken into account in order to describe obesity surgery patients’ overall level of functionality, a likely proxy of future outcome after surgical intervention. In this study, we investigate the relationship between the overall individual availability of psychological resources, depression, eating behavior and body mass index (BMI) in obesity surgery candidates.

The aim of our study is threefold; first, we want to characterize the state of psychological resource availability in patients undergoing obesity surgery.

Second, we aim at quantifying and exploring the relationship between psychological resources, depression, eating behavior, and BMI. The whole concept of psychological resources revolves around buffering or counteracting psychopathology, so that we expect a negative association between depression and availability of resources. Based on studies and meta-analyses (29, 30) suggesting a bidirectional link between depression and obesity, we assume a positive relationship between depressive symptoms and BMI. The role of eating behavior is, comparable; here, dysfunctional eating behavior increases weight gain resulting in a higher risk of depression. Finally, we test whether psychological resources could plausibly affect BMI directly, or indirectly *via* distinct pathways. Due to its theoretical and empirical dominance in the mental health sciences, we expect to observe a direct negative correlation between psychological resources and BMI.

## Methods

### Procedure

In a cross-sectional study consecutive obesity surgery candidates were assessed for a period of 20 months (from 01/2018 to 09/2019). Each patient filled out a battery of questionnaires, and participated in a semi-structured interview following a standardized medical history form afterwards. In the semi-structured interview participants were asked about weight history, previous dietary attempts, eating behavior, structure and disturbed eating behavior, psychological status, and biography. Weight and height was measured by staff.

The primary inclusion criterion was a BMI ≥ 35 kg/m^2^. Patients with substantial language barriers were excluded. The diagnoses of somatic comorbidities were taken from the consultation documentation. This study was conducted in accordance with the recommendations of good clinical practice. It was approved by the ethics committee of the medical faculty of the University of Duisburg-Essen (Ethic Vote No. 18-8450-BO). Written informed consent was given by all participants.

### Instruments

In order to measure the patients’ state of resources, their eating habits and potential depressive symptoms, the following questionnaires in paper-and-pencil form were presented to all participants. After completing the questionnaires, the patients underwent a semi-structural clinical interview following a standardized medical history form.

#### Essen Resource Inventory (ERI)

The ERI allows for a broad and nuanced measurement of a person’s resource availability. It includes 38 items. In detail, it concerns three resource areas: personal resources (e.g. self-efficacy, self-acceptance, meaningfulness, openness/flexibility, positive handling of offenses, and closeness to nature), social resources (e.g. positive social ties, practical support), and structural resources (e.g. financial security, environmental support). By its concept, all three dimensions interact and can influence each other. Here, we will only look at the global ERI-score (23).

#### Eating Disorder Examination–Questionnaire (EDE-Q)

The EDE-Q is an internationally well-established self-report questionnaire that allows a comprehensive assessment of eating behavior (31). It was developed to facilitate measurement of eating behavior, while sharing the advantages of the EDE, a clinical interview for assessing intensity of food- and weight-related worries, as well as self-restraint in eating behavior. It is considered the method of choice for eating disorder diagnosis and assessment (31–33). The EDE-Q measures specific eating disorder psychopathology by using similar operational definitions and time frames on four subscales (restraint scale, eating concern scale, weight concern scale, shape concern scale), and allows the assessment of diagnostically relevant behavioral features, for example binge eating, laxative misuse, excessive exercising, and fasting.

#### Patient Health Questionnaire (Depression Module, PHQ-d)

The PHQ-d economically measures the severity of individual depressive disorders using nine symptom-related items symptoms, in five parts. A sum score between 0 and 4 indicates no presence of depressive symptoms, 5–9 minimal depressive symptoms, 10–14 mild depressive symptoms, 15–19 moderate depressive symptoms, and 20–27 suggests severe depressive symptoms.

#### Semi-Structural Clinical Interview for Assessment of Mental Health of Obesity Surgery Candidates

This study took place in the context of pre-surgery evaluation semi-structural clinical interviews [self-made, following the general guidelines of psychosomatic evaluation interviews for obesity surgery candidates (11)], in which obesity surgery candidates are evaluated for their weight history, mental health, eating behaviors, and social support systems. Here, we will only report outcomes of mental health assessments (see **Tables 1A, B**).

**Table 1:** Sample demographics.

**1A.**			
	
Sex			
Male		n = 37 (29.13 %)	
Female		n = 90 (70.87 %)	
	
Age (years)			
Male		M = 44.24, SD = 10.83	
Female		M = 37.88, SD = 10.32	
	
Weight (kg)			
Male		M = 165.96, SD = 30.86	
Female		M = 137.36, SD = 24.81	
	
Height (cm)			
Male		M = 179.97, SD = 7.78	
Female		M = 166.29, SD = 6.67	
	
BMI (kg/m²)			
Male		M = 51.27, SD = 9.31	
Female		M = 49.26, SD = 8.22	
**1B.**			
		
Education Level (%)			
	Secondary School Leaving Certificate		34.4
	General Secondary School Leaving Certificate		34.4
	Subject-Related/General Qualification for University Entrance		15.2
	Completed College Degree		8.8
	Still In Education		0.8
	Other		6.4
		
Marital Status (%)			
	Married		37.7
	Single		33.3
	In a relationship		14.3
	Divorced		7.1
	Seperated		4.0
	Windowed		1.6
		
Psychiatric Diagnoses (ICD-10) (%)			
	Depression		
	• No depressive symptoms		3.9
	• Minimal depressive symptoms		31.5
	• Mild depressive symptoms		30.5
	• Moderate depressive symptoms		18.9
	• Severe depressive symptoms		13.4
		
	Others		
	• Binge Eating Disorder		11.8
	• Other Eating Disorder		3.9
	• Anxiety		2.4
	• Trauma		2.4

Table 1A. describes the main weight-relevant participant variables gender, age (years), weight (kg), height (cm), and body mass index (kg/m²). Table 1B shows sample characteristics concerning our participants’ education, marriage status, and prevalence of psychiatric diagnoses based on ICD-10.

### Statistical Analyses

All statistical analyses were performed in IBM SPSS Statistics (Version 25) and R3.6.1 [R Core Team (34)]. The level of significance for all analyses was set at α = .05. Gaussian normality was assessed visually using qq-plots (see **Supplementary Material**). The patients’ BMI indeed deviated heavily from normality, so it was log-transformed in order to ensure normality approximation. We further complemented our results by using robust estimators to test if our results were heavily biased by normality violations (see **Supplementary Material**). Indeed, the overall pattern remained similar here.

We, first assess the direct impact of eating behavior, depression, and psychological resources on the patients’ weight. To do so, we constructed a multiple regression model with the PHQ-d, the EDE-Q, and the ERI predicting the patients’ BMI. Psychological resources as such did not directly affect the BMI, however, we then applied path modeling in an exploratory fashion [using the R-package *Lavaan*, (35)] to evaluate whether there still might be a plausible directional effect of ERI on participants’ weight. In other words, we investigated whether the effect of resources on weight could be mediated *via* depression and eating behavior. In order to evaluate model fit and parsimony, we used the comparative fit index (CFI, cut-off ≥.90), the Tucker Lewis Index (TLI, cut-off ≥.90), the root mean square error of approximation (RMSEA, cut-off ≤.08), and the standardized root mean square residual (SRMR, cut-off ≤.08) with cut-offs as described in Gana and Broc (36). Please note that such path models**—**in the way we use them—cannot inform us about, nor test the underlying causal structure between the variables given our cross-sectional design.

## Results

### Sample

A total of 127 patients participated in the study, with 90 female (70.87%) and 37 male obesity surgery candidates. The average age was 39.73 (SD = 10.82, range = 19–67), with men being on average 44.24, and women 37.88 years old. The selection criterion was BMI ≥ 35 kg/m² (range 36.7–84.2 kg/m²) Descriptive statistics are shown in **Tables 1A, B**.

### Effects of Psychological Resources on BMI

In order to assess the direct effect of psychological resources, depression severity, and eating behavior on the patients’ BMI before surgery, we regressed ERI, PHQ-d, and EDE-Q on the log-transformed BMI using ordinary least squares linear regression. Overall, no critical multicollinearity occurs (with all Variance Inflation Factor (VIF) < 2.0) and the model explains 10.5% of all variance (with an adjusted R² = .083). Adding all higher order interaction terms does not significantly reduce residual sum of squares (p = .936). As shown in **Table 2**, and in conformance with our hypotheses, the PHQ-d indeed shows a positive association with BMI. Also, we find a negative relationship between overall eating behavior**—**such as weight and shape concern and restrictive eating**—**with the patients’ BMI. Hence, patients who report to restrain themselves and to be concerned about their weight, shape, and body are overall less overweight. Contrary to our hypotheses, availability of psychological resources**—**measured by the ERI**—**does not predict BMI; here, we find a slightly positive, but non-significant β-estimate. Additional exploratory analyses show that, indeed, even when quartile-splitting ERI and comparing participants in the lowest vs the highest quartile on their BMI, we find no difference (t (30) = −.400, p = .621). Hence, there is no evidence for a directly modulating role of psychological resources on the patients’ weight.

**Table 2:** Regression analysis to predict the BMI using Patient Health Questionnaire (PHQ-d), Eating Disorder Examination-Questionnaire (EDE-Q), and Essen Resource-Inventory (ERI).

Predictor	β	βse	t	p
Intercept^a^	0.006	0.085	0.065	0.948
EDE-Q^a^	−0.357	0.101	−3.522	0.001
PHQ-d^a^	0.313	0.115	2.718	0.008
ERI^a^	0.116	0.102	1.131.	0.26

Total R² = .105 (N = 127, p = .004).

^a^All variables are standardized.

### Path Model Analyses

As shown, psychological resources do not influence BMI directly. However, resources might still exert an indirect effect on patients’ pre-surgery weight. In order to explore such *potentially plausible* causal pathways between ERI and BMI, we constructed two exploratory path models evaluating if depressive symptoms and eating behavior mediate such connection; here, the effective direction leads from the ERI and reaches BMI *via* PHQ-d and EDE-Q.

First, we investigated if ERI might indirectly exert its effect on BMI *via* the PHQ-d and the EDE-Q at the same time. We modeled paths from the ERI to BMI directly, from ERI to PHQ-d, from ERI to EDE-Q, as well as form PHQ-d and ERI to BMI (see **Supplementary Material** for illustration). Indeed, the path estimates reveal that such two-armed mediation might take place, and that ERI might reduce the likelihood of high BMI *via* downregulation of PHQ-d, on the one side, but on the other side, increase BMI *via* the upregulation of more controlled eating behavior (see **Supplementary Material**). Yet, this model showed a subpar fit to our data with a CFI of .701 and a RMSEA of .468. Also, a significant χ²-test (χ²(1) = 28.326, p <.001) indicates that the observed correlation matrix could not be properly reproduced by the model.

**Figure 1:**
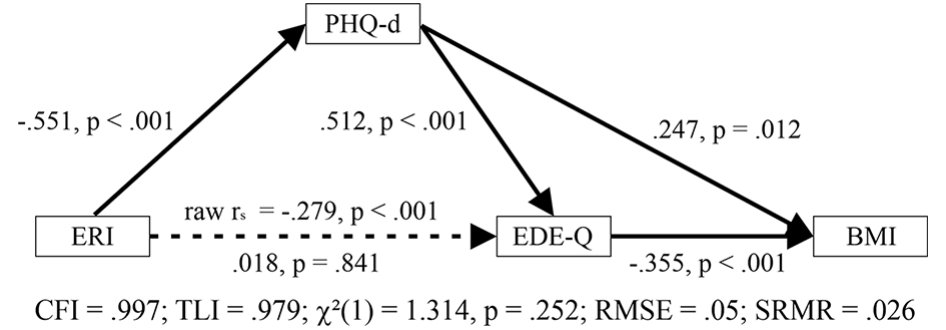
Path model describing the relationship between psychological resources (ERI) and BMI. Here, the influence of psychological resources is completely mediated *via* depression (PHQ-d), which, on the one hand, directly impacts the BMI positively, and, on the other hand, mediates it *via* increasing restrictive eating behavior. The original negative association between ERI and EDE-Q is completely mediated by PHQ-d.

Next, we examined in a new model whether the influence of psychological resources on eating behavior as such is actually produced by its impact on PHQ-d: Depression, a variable that is heavily influenced by the extent of overall psychopathology, is very central in predicting a vast number of dysfunctional, or restrictive and worrying behaviors. To examine such mediation effect, we added an additional path from the PHQ-d to the EDE-Q (see **Figure 1** and **Table 3**); again, ERI might only exert its modulatory role on eating behavior *via* the PHQ-d. To gain additional degrees of freedom and allow for model convergence, we removed the direct path between ERI and BMI, for which we know that there is no relationship. Indeed, the path estimate between ERI and EDE-Q does not significantly predict EDE-Q anymore. The effect of psychological resources on eating behavior is fully mediated by depression. As before, two effective pathways appear *via* which the ERI might affect the patients’ weight, but this time, they origin from the PHQ-d. According to this model, ERI downregulates PHQ-d. From there depression seems to have a downregulating effect. On the other hand, PHQ-d may affect dysfunctional eating behavior positively, which is associated with a reduced BMI. According to this model, there might be potentially plausible causal connections between resources and the patients’ weight; these connections, however, do not manifest in a direct correlation between ERI and BMI because they might cancel each other out. Importantly, the model shows acceptable fit indices with a CFI of.007, a TLI of.979, a RMSEA of.05 (CI [0:.25]), a SRMR of.026. Also, the χ²-test assessing whether the deviation between the observed and reproduced correlation matrix is critically high is non-significant, indicating a good model performance (χ²(1) = 1.314, p = .252). The same pattern of path estimates emerges when applying a more robust estimator, too (see **Supplementary Material**). 

**Table 3:** Path coefficients for our main model.

Path	Std. Coefficient	Coeffse	z	p
ERI → PHQ-d	−0.551	0.074	−7.43	<.001
ERI → EDEQ	0.018	0.09	0.201	0.841
PHQ-d → EDEQ	0.512	0.091	5.638	<.001
PHQ-d → BMI	0.247	0.098	2.514	0.012
EDE-Q→BMI	−0.355	0.1	−3.541	<.001
χ²(1) = 1.314, p = .252; CFI = .997, TLI = .979; RMSEA = .05, CI [0:.250]; SRMR = .026				

All path coefficients differ significantly from zero, except the association between psychological resources (ERI) and dysfunctional eating (EDE-Q). This relationship is fully mediated by depression. Fit indices indicate an acceptable fit to our data with a CFI and a TLI >.90, and a RMSEA and SRMR <.08. Also, the χ²-test is non-significant.

## Discussion

To our knowledge, this is the first study exploring the impact of psychological resources on BMI in obesity surgery candidates, even though these have been found to be a major contributor to a variety of psychopathological domains, and largely predictive of health behavior in somatic diseases. We therefore hypothesized that psychological resources also affect BMI directly or mediated by depression and/or eating behavior in obesity surgery candidates.

Our findings showed that psychological resources do not have a direct influence on BMI, but seem to have a mediating effect on weight through depression and eating behavior. We found no significant direct correlation between psychological resources (overall ERI-score)—measuring personal, as well as structural and social psychological resources**—**and the BMI. To determine mediating effects of depression and eating behavior path models were constructed and selected based on model fit.

The final model reveals two pathways *via* which psychological resources could have a mediating effect on BMI: here, psychological resources do not exert any direct effect on eating behavior, nor BMI itself, but they are associated with an attenuated severity of depression symptoms, as measured by the PHQ-d. From there, the effect on BMI seems to be bidirectional: on one side, depression severity seems to be directly associated with BMI, but also might influence increasing worries about eating, weight, shape, and eating restraint, which, on the other side, are associated with a BMI increase. Although we find raw correlations between psychological resources and eating behavior, this effect seems to be fully mediated by depression. Hence, the effect of psychological resources on weight seems to be mediated by depressive symptom severity, which might influence the BMI increasingly and decreasingly at the same time.

The positive association between depression and BMI is not surprising, given that depression and obesity are strongly intertwined (13, 29). This is particularly intriguing in patients with the prospect of obesity surgery: Approximately 66% of patients eligible for surgery were found to suffer from at least one psychiatrically relevant condition once in their lifetime, with 38% meeting the criteria for an axis I diagnosis for DSM-IV at the time of the surgery (22). Our path analysis reveals that, on the one side, there is direct positive association between depression and BMI. This is in line with previous literature that has also suggested causal pathways *via* which depressive patients gain weight (37).

It is also not surprising that psychological resources are associated with decreasing psychopathological symptoms given the literature on the stress-buffering effect of resilience, social and structural support, and personal capacities (25, 26). Literature on the positive association between positive and negative traits is rare (38, 39). Therefore, we could assume at this point that psychological resources alleviate depressive symptoms and thus have a decreasing potential for the risks of weight gain. On the other side, we find that severity of psychopathological symptoms could have an enhancing effect on restrictive eating and worries about body weight and shape, as measured by the EDE-Q. This seems to be counterintuitive at the first glance, especially considering that high dysfunctional restrictive eating and weight worrying behavior in community samples predicts BMI positively**—**thus, the more people seem to restrain themselves and worry about their body shape, the more they actually may weigh in community and underweight samples.

Still, it needs to be considered that our sample is highly selective, with obesity surgery mostly being the last resort for patients with an already exhausted set of conservative attempts to lose weight. In these, mostly severely obese patients, any restriction or worry yield benefits for maintaining weight levels, even if they go along with higher psychological burden that cause the restraint (33). Thus, our second pathway shows that psychological resources, might influence the severity of depressive symptoms decreasingly and diminish a focus on eating restriction and worries about patients’ own body. This would, indeed, lead to an increase of the BMI due to the presence of psychological resources.

Our sample is highly representative revealing similar characteristics as presented in other studies: The global EDE-Q norms provided for obese females without an eating disorder in the study by Aardoom et al. (31) showed similar characteristics regarding their eating behavior (global EDE-Q score in this study M = 2.75 vs. in our study M = 2.676, t(126) = −0.786, p = 0.433), whereas they find higher EDE-Q scores in binge-eating, but lower ones in non-obese healthy participants [see also Rø et al. (32)]. Also, our samples’ BMI fall within the range of the participants recruited in other studies, although our participants are, on average, slightly more obese. To illustrate: the sample surveyed in this study had a mean BMI of 49.85, while the samples examined by Gero, et al. (33) or by Çalışır et al. (40) had a slightly less obese BMI with a mean BMI of 43.97 and 47.26, respectively.

Still, patients with obesity without an eating disorder also score higher on global EDE-Q, as well as on its subscales (e.g. weight, shape concern) than the norm (32). Concerning our sample, this suggests the conclusion, that the patients try to lose weight and pay more attention to eating and body image in general.

However, our sample as such is of moderate size, and our model needs to prove robust on a larger group of patients in order to make significant presumptions. Furthermore, an increase in sample size would allow us to test a larger variety of path-model alternations, including residual variances between unconnected variables, as well as potential latent variables. Although we consider it unlikely—given the importance of psychological resources in psychological and somatic disease management—we must take into account (following a more conservative interpretation of mediation statistics) the possibility that psychological resources show no significant relationship with obesity, or that (for instance endophenotypical) latent factors carry the primary causal role for depression, lack of resources, and in turn obesity. Our cross-sectional approach precludes any statements about the directional causal structure of the variables. For instance, longer-lasting depression might have already lead to a fundamental decay in self-regulation and functional coping mechanisms, but also to social withdrawal, decline and economic deprivation, and thus, might have critically reduced psychological resources. Hence, depression might have been more central in our path model in that it could be the main reason for diminishing psychological resources instead of the other way around. Although we believe that psychological resources are by all means essential to the overall capability to cope with adversarial life circumstances**—**as shown by a large number of intervention and longitudinal studies (e. g. on the effectiveness of psychotherapy or community psychiatry programs specifically aiming at increasing psychological resources) our design does not illuminate, or cannot distinguish between, causal directions. Yet, a recent paper by Lund and Cois (41) could show in a longitudinal design using economic income measures, which could be considered a proxy for at least social or structural resources, that independent time-lagged effects of depression on income, as well as vice versa, exist. Indeed, a look at the regression coefficients reveals that the effect of income on (time-lagged) depression is larger than depression on income. Also, it needs to be mentioned that, whether personal resources or coping mechanisms primarily affect psychopathological states or vice versa is one of the key questions in the mental health sciences.

In this study, depression was used as the main point for the analysis, but not on other psychiatric diseases. This was planned because depression is very central in people suffering from obesity (13, 29). Of course, this limits the scope of this study, and follow up studies should quantify the severity of other psychiatric diseases. In the same line, the possibility of residual confounding cannot be excluded. For instance, it is known that there is a non-negligible association between early life stress and adverse life events and psychiatric conditions, such as aberrant eating behavior (42) and depression (43). Knowing about early life stress could, indeed, contribute to drawing a more complete picture about the association between depression, resources, and dysfunctional eating behaviors. In a similar fashion, ostracism and stigma might be strongly intertwined with depression and obesity (44).

Moreover, it remains to be mentioned that the EDE-Q was originally designed to measure dysfunctional eating behaviors, and is supposed to use to rather interpretative than to categorize—this subpopulation actually has a lower BMI when having high EDE-Q values. Here, the EDE-Q might have indeed measured more functional, healthy restraint eating behavior instead of dysfunctional eating and has only limited pathological value. Additionally it needs to be considered that our data (regarding resources, eating behavior and depressive symptoms) is based on self-reported information and was collected simultaneously. Therefore, there is a considerable risk of bias due to common method variance.

Lastly, longitudinal, as well as interventional studies with varying support- and treatment-interventions targeting cultivation of psychological resources and patients’ motivation would allow for proper and controlled causal inference. Thus, they could shed light on mechanisms and therapy-components that could indeed improve the patients’ well-being and elevate their success in long-term weight loss. This would pick up on the findings of de Zwaan et al. (13) which showed that it is important to detect depressive disorders in obese patients, although depression significantly decreased after obesity surgery, as this can have a negative impact on postoperative success. This is also in line with the suggestion of Kalarachian et al. (45) that patients with known present or past psychological disorders should be monitored closely to support improved short- term surgical outcomes. Studies focusing on post-operative treatment addressing e.g. depression do exist: Wild et al. (46, 47) showed, that an 1 year aftercare program after surgery leads to improved self-efficacy and less depressive symptoms in a 4 year follow-up. These findings indicate the importance of supportive treatment to foster psychological resources, especially for long-lasting reduction of patients’ weight and improvement of overall well-being.

Taking individual psychological profiles of obesity surgery candidates into account and therefore taking a closer pre-surgery look could help to single out risk patients and make sure that they get a proper treatment before and after the surgery to ensure better long-term success in weight-loss and maintenance (48). In terms of even more improved success, the richness of the tradition of positive psychology could be fully exploited, and even more positive traits and factors could be taken into account and fostered in a more specific and differentiated manner (see e.g. Garaigordobil (28) for an investigation of a link between self-esteem, happiness and psychopathology). It remains, however, noteworthy, that although positive psychology has had a massive increase in popularity during the recent years, not all interventions appear to yield appropriate effects (38, 39). Also, contextual factors may not be dismissed when defining whether characteristics and behaviors are *positive* (49).

To summarize and conclude: The model suggests a dichotomous, bidirectional effect of psychological resources on BMI: again, ERI seems to buffer negative direct effects of depression on weight, but indirectly also have an decreasing effect on self-restriction and the, although worrying, focus on the own body, which could attenuate overeating wherever possible. This twofold effective direction could, indeed, explain why we find no direct relationship between psychological resources and the patients’ BMI; personal capacity to deal with difficulties, as well as structural and social embeddedness, have both positive and negative effects on BMI, but they might cancel each other out.

If these assumptions prove solid, this would have remarkable implications for day-to-day interdisciplinary care and support of obesity surgery patients as well as on a supposedly overall improved surgical outcome. By solely attempting to reduce depressive symptom severity, e.g. by reducing chronic distress, one could diminish the risk of gaining more weight, but also decrease the chances of restricting behavior. Therefore, it is important to provide patient support to improve psychological resources in the context of obesity surgery. Like mentioned above, Wild et al. (46, 47) examined the positive influence of patients’ support systems (e.g. psychological resources) after surgery and found that psychoeducational intervention shows sustained effects on both depression severity scores and self-efficacy.

Regarding this aspect our findings take the next step and therefore build a foundation to further understanding intervention-efficacy in the obesity surgery context.

Our results could prepare further investigations on how to offer the best possible resource-based psychological and psychosomatic support.

## Data Availability Statement

The datasets generated for this study are available on request to the corresponding author.

## Ethics Statement

The studies involving human participants were reviewed and approved by Ethics Committee of the medical faculty of the University of Duisburg-Essen. The patients/participants provided their written informed consent to participate in this study.

## Author Contributions

AR, AS, MH, MN, ND, SH, TH, ST, MT, and E-MS contributed to the conception and design of the study. AR, E-MS, and ND substantially contributed to the acquisition of data for the study. AS and MH performed the statistical analysis. AR wrote the first draft of the manuscript. All authors contributed to the article and approved the submitted version.

## Funding

We acknowledge support by the Open Access Publication Fund of the University of Duisburg-Essen.

## Conflict of Interest

The authors declare that the research was conducted in the absence of any commercial or financial relationships that could be construed as a potential conflict of interest.
