# Supplementary material for: The Impact of Psychological Resources on Body Mass Index in Obesity Surgery Candidates
Source: Front Psychiatry. 2020 Jul 10;11:649. doi: 10.3389/fpsyt.2020.00649 (PMC7365886; doi:10.3389/fpsyt.2020.00649)
Supplement: Supplementary file 1 [file DataSheet_1.pdf]

Supplementary Material

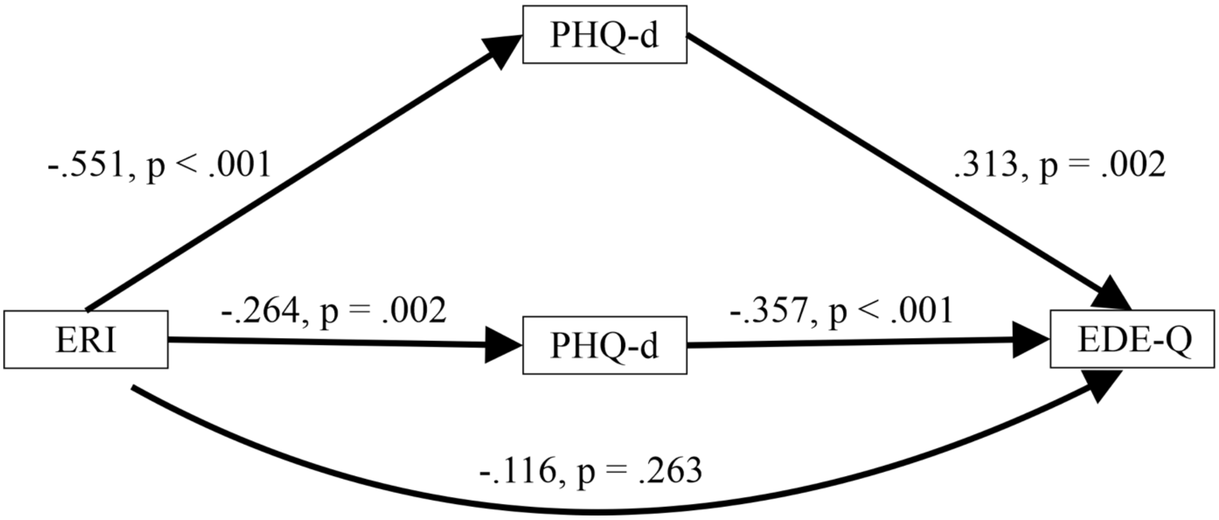

Supplementary Figure 1. First mediatory path model. Although resources (ERI) might exert a potential influence on BMI via depression (PHQ-d) and eating behavior (EDE-Q) based on the path coefficients, the overall model fit is subpar with a low CFI value, a significant χ^2-test and a high RMSE and SRMR. ($\chi^2(1) = 28.326$, $p < .001$; CFI = .701, RMSEA = .468, CI [.329:.623]; SRMR = .129).

Robust Path Models

When determining and evaluating coefficients in a path or structural equation model, statistical inferences based on a maximum-likelihood estimator very prone to being biased by violations of Gaussian normality (36). In our sample variables approximate normality, as evaluated in qq-Plots (see Supplementary Figure 2), but are not perfectly normal. For instance, according to Shapiro-Wilk-Tests, only the distribution of the EDE-Q reaches the criterion for normality, although caution is required because some normality tests become very sensitive towards small deviations of normality with higher sample size (50). The R-package lavaan offers a number of more robust estimators in case of violated normality or homogeneity of variance. Here, the MLM-estimator includes robust standard errors and a Satorra-Bentler χ^2 scaling correction (35, 36) Similarly, the MLR-estimator uses a Satorra-Yuan χ^2, more appropriate for smaller sample sizes, and Huber-White robust standard errors (35).

A recomputation of our main path model using these two estimators reveals a very a similar pattern compared to reported model with ordinary ML-estimators, as well as to each other (see supplementary table 1). Hence, a potential normality violation in our data appears to be minor importance here.

Model	Path	Std. Coefficient	$Coeff_{se}$	z	p
MLM Estimator	ERI → PHQ-d	-.551	.070	-7.827	< .001
	ERI → EDEQ	.018	.093	.195	.845
	PHQ-d → EDEQ	.512	.095	5.394	< .001
	PHQ-d → BMI	.247	.103	2.399	.016
	EDE-Q → BMI	-.355	.103	-3.485	< .001
	$\chi^2_{scaled}(1) = 1.678$, p = .195; CFI_{scaled} = .993, $RMSEA_{scaled}$ = .074; SRMR = .026				
MLR Estimator	ERI → PHQ-d	-.551	.070	-7.827	< .001
	ERI → EDEQ	.018	.093	.195	.845
	PHQ-d → EDEQ	.512	.095	5.394	< .001
	PHQ-d → BMI	.247	.103	2.384	.017
	EDE-Q → BMI	-.355	.104	-3.485	< .001
	$\chi^2_{scaled}(1) = 1.934$, p = .164; CFI_{scaled} = .990, $RMSEA_{scaled}$ = .086; SRMR = .026				

Supplementary Table 1. Main path model recomputed with robust standard errors and scale-corrected β^2-tests (MLM and MLR-estimators in lavaan).

Model	Path	Std. Coefficient	$Coeff_{se}$	z	p
1	ERI → PHQ-d	-0.551	0.074	-7.43	< .001
	ERI → EDEQ	-0.264	0.084	-3.13	0.001
	PHQ-d → BMI	0.313	0.101	3.094	0.002
	EDE-Q → BMI	-0.357	0.089	-4.009	<.001
	ERI → BMI	0.116	0.103	1.12	0.263

$\chi^2(1) = 28.326$, $p < .001$; CFI = .701, TLI = -.795, , RMSEA = .468, CI [.329:.623]; SRMR = .129

Supplementary Table 2. First mediation model with no pathway between dysfunctional eating and depression (see supplementary figure 1). Here, fit indices clearly indicate subpar overall fit to the data.

Robust Multiple Regression

As mentioned above, linear models might be prone to bias if normality is violated. Thus, results of our multiple linear regression, assessing the direct impact of the PHQ-d, the ERI and the EDE-Q might also be biased due to slight deviation from a normal distribution. Multiple regression can also be computed with Huber-White robust standard errors. Here, robust standard errors do also not have a strong impact on the outcome. There is still a significant positive direct association between PHQ-d and BMI (β = .313, βse = .120, z = 2.605, p = .009), and the association between EDE-Q (β = -.357, βse = .101, z = -3.530, p <. 001) and BMI remains significant, as well. Also, the relationship between ERI and BMI remains non-significant (β = .116, βse = .088, z = 1.314, p = .189). Again, the slight violation of normality does not have significant impact on our main results.

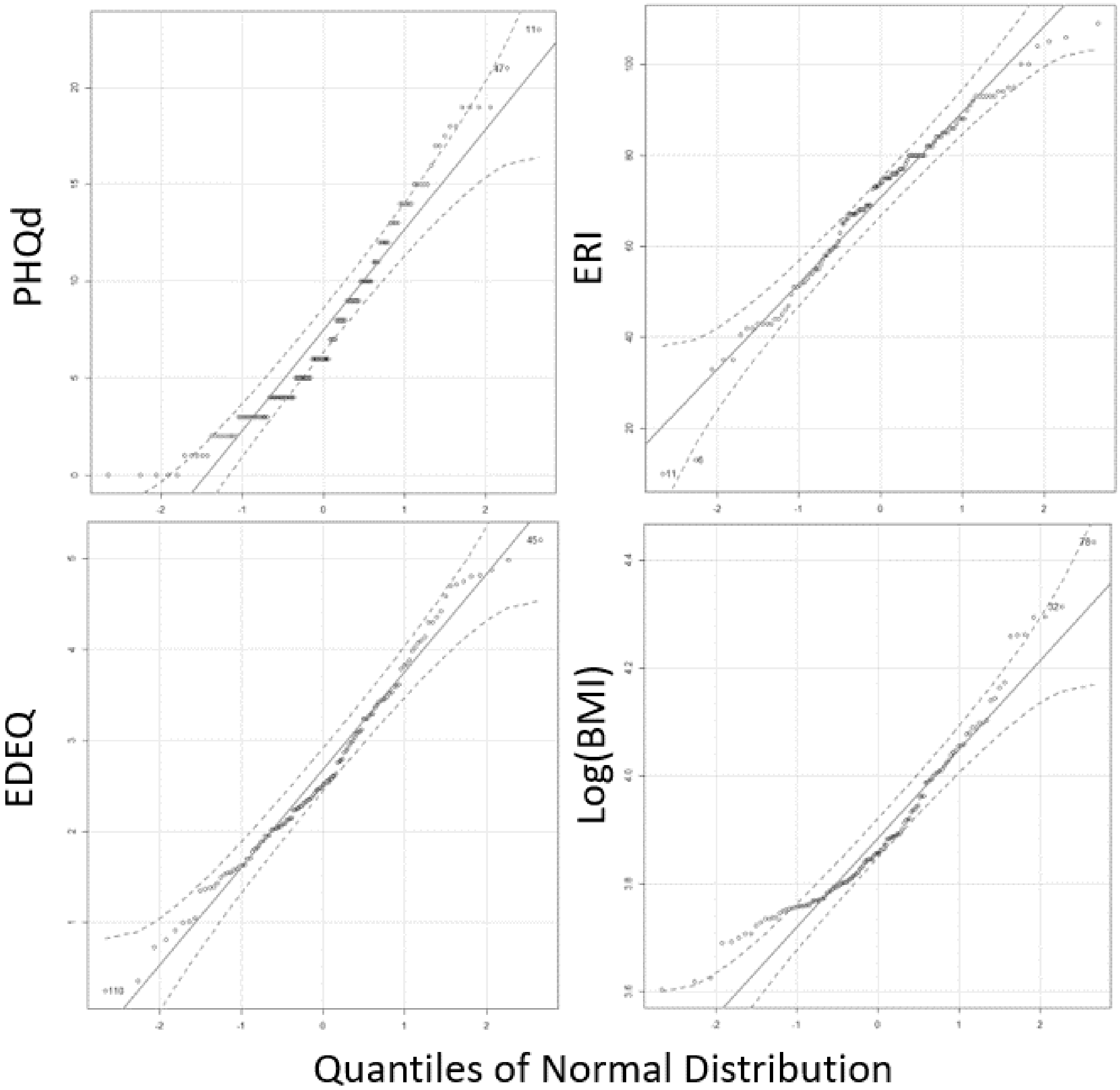

Supplementary Figure 2. qq-Plots of our main variables. Individual variable distributions were plotted against a perfect normal distribution to evaluate normality. All variables approximate normality, although violations are particularly observable in the PHQ-d, as well as the logarithm of the BMI in spite of the transformation. Note, however, that these violations did not affect our results when robust estimators were applied.

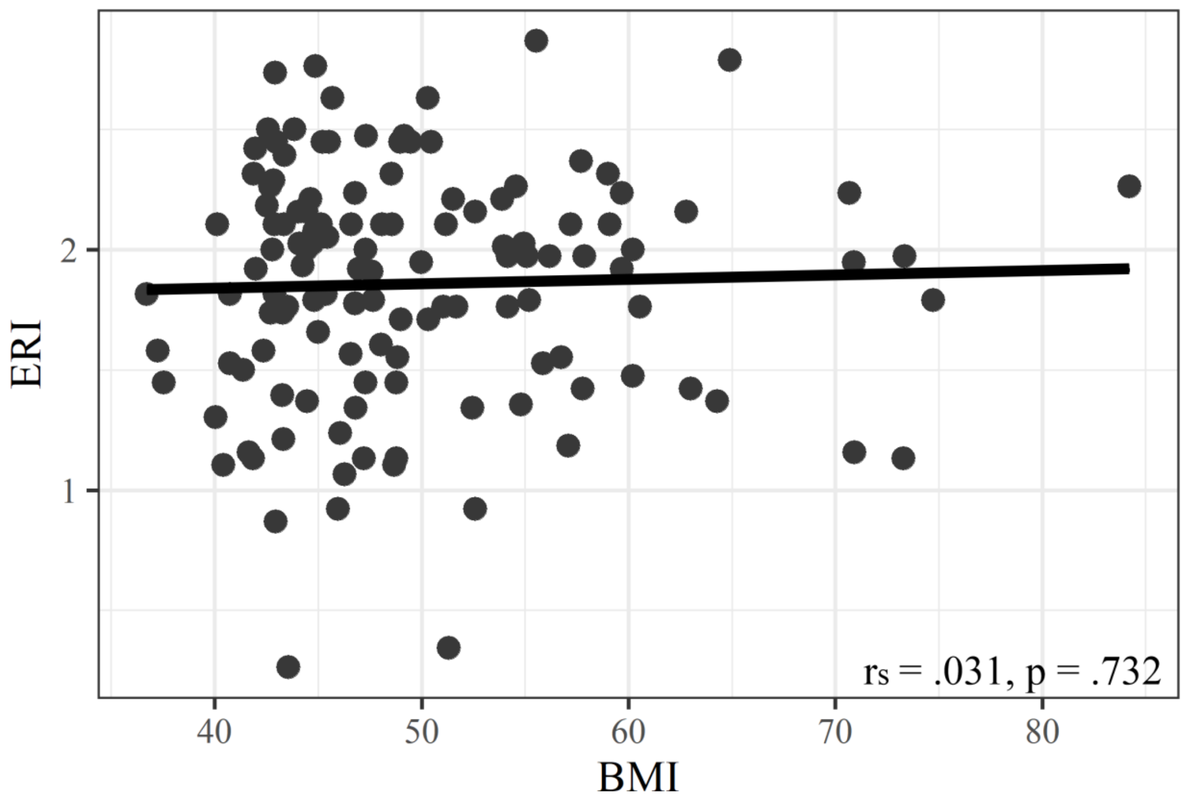

Supplementary Figure 3. Scatterplot of correlation between psychological resources (ERI) and BMI. There is no significant association between resources and weight at the time point of measurement.

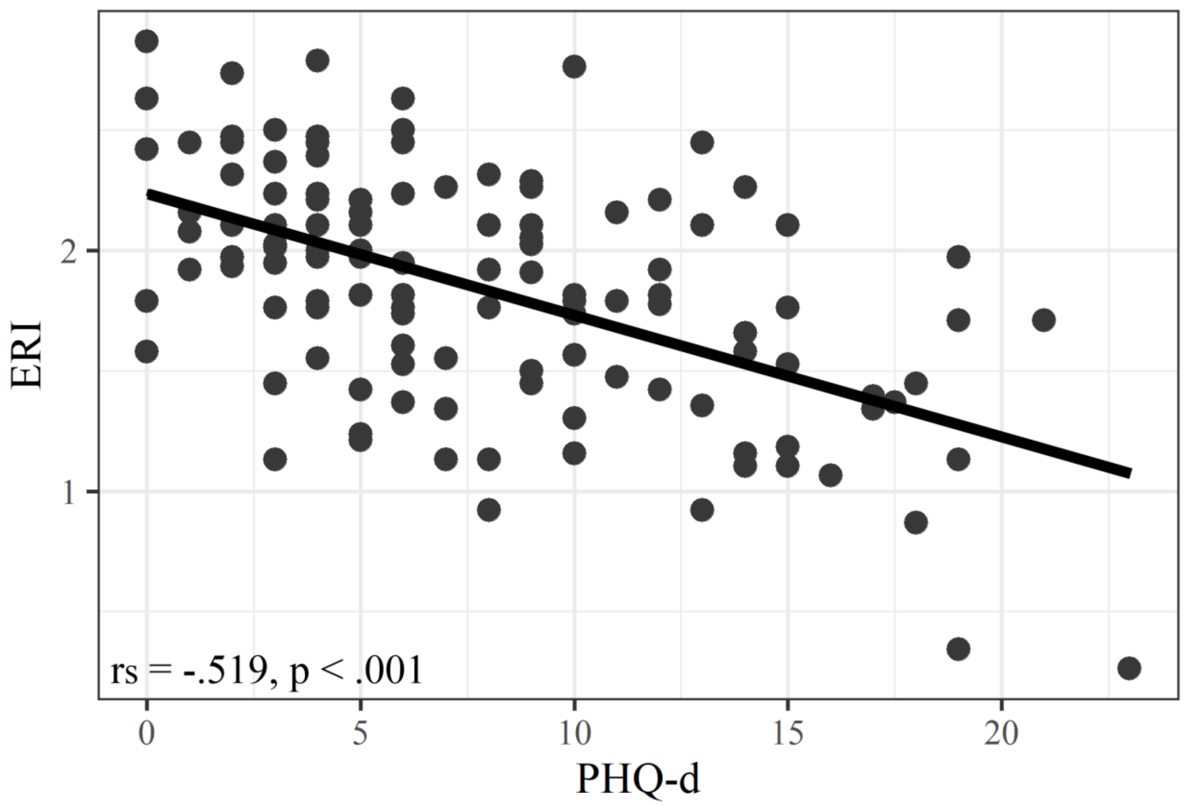

Supplementary Figure 4. Scatterplot of correlation between psychological resources (ERI) and depressive symptom severity (PHQ-d). There's a negative association between psychological resources and depression.

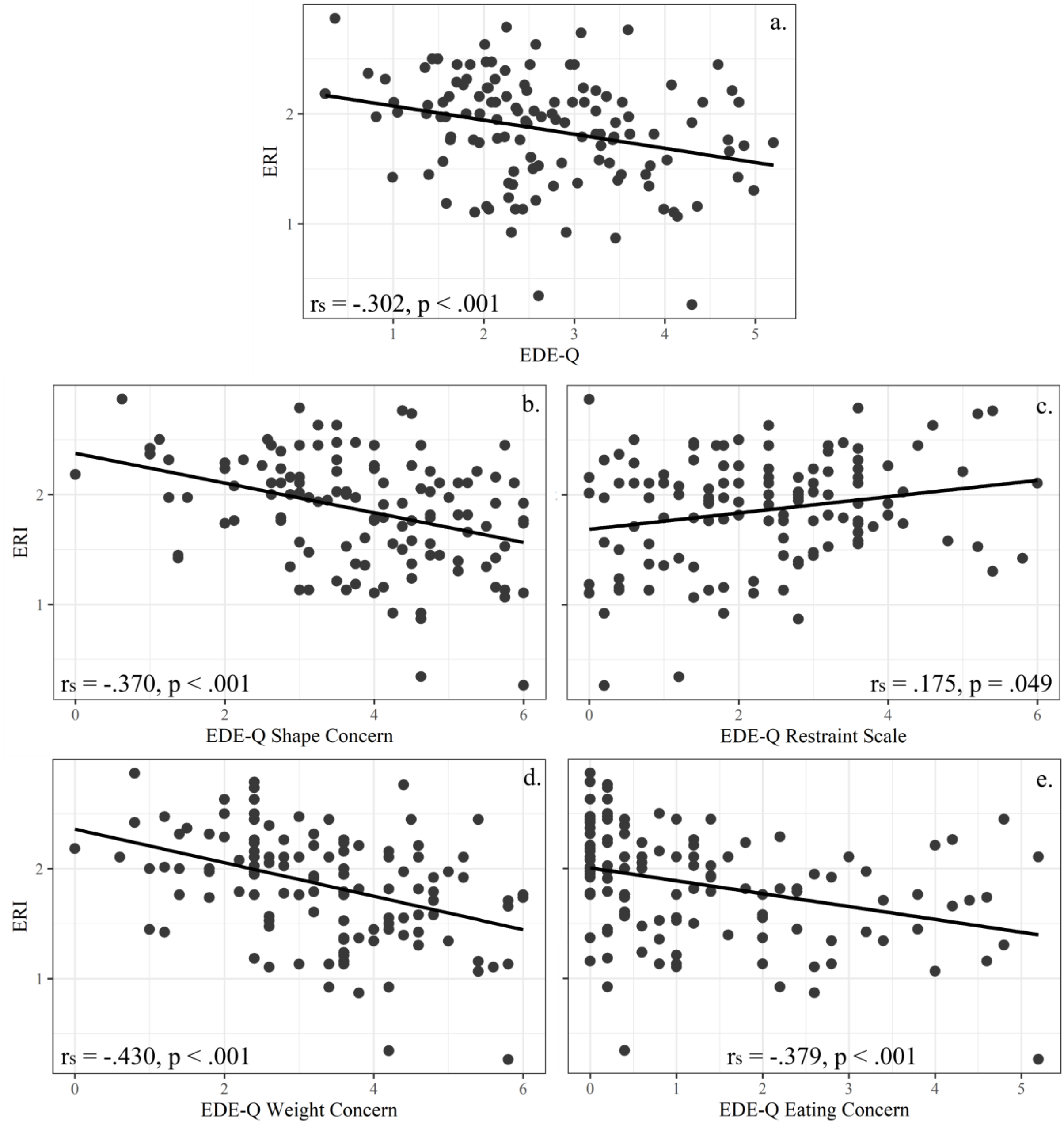

Supplementary Figure 5. Scatterplots depicting the association between dysfunctional eating behaviors (EDE-Q) and psychological resources (ERI). In this specific sample of obesity surgery candidates, we find a negative relationship between psychological resources and dysfunctional eating behavior. Psychological resources are associated with increasing restraint behavior (see panel C), but do not severely affect the overall score.
